# Effect of Central Injection of Neostigmine on the Bacterial Endotoxin Induced Suppression of GnRH/LH Secretion in Ewes during the Follicular Phase of the Estrous Cycle

**DOI:** 10.3390/ijms20184598

**Published:** 2019-09-17

**Authors:** Andrzej Przemysław Herman, Janina Skipor, Agata Krawczyńska, Joanna Bochenek, Karolina Wojtulewicz, Bartosz Pawlina, Hanna Antushevich, Anna Herman, Dorota Tomaszewska-Zaremba

**Affiliations:** 1 The Kielanowski Institute of Animal Physiology and Nutrition, Polish Academy of Sciences, 05-100 Jabłonna, Poland; agata_krawczynska@wp.pl (A.K.); jb.tst@wp.pl (J.B.); k.wojtulewicz@ifzz.pl (K.W.); b.pawlina@ifzz.pl (B.P.); a.antuszewicz@ifzz.pl (H.A.); d.tomaszewska@ifzz.pl (D.T.-Z.); 2 Institute of Animal Reproduction and Food Research, Polish Academy of Sciences, 10-748 Olsztyn, Poland; j.skipor@pan.olsztyn.pl; 3 Faculty of Health Sciences, Warsaw School of Engineering and Health, 02-366 Warsaw, Poland; anna.herman@onet.pl

**Keywords:** inflammation, cytokines, neostigmine, GnRH, luteinizing hormone, reproduction disorders

## Abstract

Induced by a bacterial infection, an immune/inflammatory challenge is a potent negative regulator of the reproduction process in females. The reduction of the synthesis of pro-inflammatory cytokine is considered as an effective strategy in the treatment of inflammatory induced neuroendocrine disorders. Therefore, the effect of direct administration of acetylcholinesterase inhibitor—neostigmine—into the third ventricle of the brain on the gonadotropin-releasing hormone (GnRH) and luteinizing hormone (LH) secretions under basal and immune stress conditions was evaluated in this study. In the study, 24 adult, 2-years-old Blackhead ewes during the follicular phase of their estrous cycle were used. Immune stress was induced by the intravenous injection of LPS *Escherichia coli* in a dose of 400 ng/kg. Animals received an intracerebroventricular injection of neostigmine (1 mg/animal) 0.5 h before LPS/saline treatment. It was shown that central administration of neostigmine might prevent the inflammatory-dependent decrease of GnRH/LH secretion in ewes and it had a stimulatory effect on LH release. This central action of neostigmine is connected with its inhibitory action on local pro-inflammatory cytokines, such as interleukin (IL)-1β, IL-6, and tumor necrosis factor (TNF)α synthesis in the hypothalamus, which indicates the importance of this mediator in the inhibition of GnRH secretion during acute inflammation.

## 1. Introduction

The host immune system developed to identify conserved bacterial molecular patterns. Among them are bacterial cell wall/outer membrane components, such as a bacterial endotoxin—a lipopolysaccharide (LPS) located in the outer membrane of Gram-negative bacteria that can be a signal of bacterial infection. Also, its role is to initiate an inflammatory response [1,2]. LPS is released from bacteria in the wake of cell division, death, or especially, antibiotic treatment against bacterial infection. It is well known that circulating LPS induces immune stress in the host organism, which may be accompanied by impaired reproductive functioning. In the organism, the ongoing inflammation influences the activity of the neurohormonal system of the hypothalamic-pituitary-gonadal (HPG) axis and in females it can lead, among others, to the disruption of ovarian cyclicity or even to the loss of pregnancy [3,4].

Although the direct modulatory action of LPS on the HPG axis activity, both, at the hypothalamic [5] and pituitary [6] levels were reported, it is rather accepted that endotoxins disturb this axis activity indirectly by the activation of inflammatory mediator production. It is widely known that pro-inflammatory cytokines, such as interleukin (IL)-1β, tumor necrosis factor (TNF)α, and IL-6 are the main mediators between the immune and neuroendocrine systems [4]. These cytokines, according to previous reports, can reach the anterior pituitary (AP) via peripheral blood or can be locally synthesized in the pituitary cells [7,8,9] and may influence gonadotropin secretion directly in the AP [10,11,12]. However, it is postulated that most immune-neuroendocrine interactions occur in the hypothalamus, which by tonic secretion of gonadotropin-releasing hormone (GnRH) to the hypothalamic hypophysial portal system, primarily controls female reproduction. Peripheral inflammatory stimuli increase not only the circulating level of pro-inflammatory cytokines but also elevate their concentration in the cerebrospinal fluid (CSF) [13,14]. Although the area of the brain is protected against an uncontrolled influx of peripheral molecules, it was previously proven that pro-inflammatory cytokines could cross the blood-brain barrier (BBB) by an active transport mechanism and could be de novo synthesized by the BBB cells [15,16]. Also, the brain’s choroid plexus (CP) epithelium, which forms the blood-CSF-barrier (BCSFB), play an important role in the communication between immune and neuroendocrine systems because LPS treatment induces the expression of pro-inflammatory cytokines and their receptors in the CP [17]. It is worth mentioning that pro-inflammatory cytokines are also considered to be important regulators of the reproduction process in the gonads and uterus. Although inflammatory cytokines regulate the endometrium functioning in healthy females, inflammation plays a crucial role in the development and progression of endometriosis as it is closely associated with the overproduction of an array of inflammatory mediators [18]. The increased gene expression of inflammatory cytokines, including IL-1β, IL-6, and TNFα in the endometrium and macroscopically normal peritoneum in women with endometriosis was reported [19,20]. Inflammatory cytokines seem to be also involved in the pathophysiology of polycystic ovary syndrome (PCOS), which is one of the most common endocrine alterations in women of reproductive age. It was found that pro-inflammatory cytokines are able to induce insulin resistance, which accompanied by hyperinsulinemia are among the main reasons of PCOS and related hyperandrogenism [21,22].

Although some amount of pro-inflammatory cytokines occurring in the central nervous system (CNS) during an inflammatory challenge could have peripheral origin or could be secreted by the CP cells, it is believed that centrally acting inflammatory cytokines are primarily locally synthesized in CNS cells. The local synthesis of pro-inflammatory cytokines in the hypothalamus of many species, including sheep was also reported [23,24,25,26,27,28]. These central pro-inflammatory cytokines of different origins may influence the neuroendocrine system functioning because their corresponding receptors are expressed not only in the region of hypothalamus but also in the structures involved in GnRH-ergic activity [24,29,30,31].

The reduction of the pro-inflammatory cytokines synthesis is often considered by researchers as an effective strategy in the treatment of inflammatory induced neuroendocrine disorders. In our previous studies on sheep, we demonstrated that the peripheral administration of systemic acetylcholinesterase (AChE) inhibitor reduced inflammatory-induced synthesis of pro-inflammatory cytokines in the hypothalamus [23,25]. It was also found that the inhibitory effect of LPS administration on the GnRH/luteinizing hormone (LH) secretion could be terminated by peripheral treatment with AChE inhibitors [29,32]. We also found that the reduction of pro-inflammatory cytokines concentration in the peripheral blood by neostigmine—an AChE inhibitor not able to cross the brain barriers—blocked the transition of the inflammatory signal into the brain parenchyma and was sufficient to reduce the synthesis of these mediators in the hypothalamus during acute immune stress [23]. Moreover, neostigmine treatment prevented inflammatory-dependent changes in GnRH/LH secretion [29]. This observation could suggest the incredibly important role of blood-born cytokines in the induction of neuroinflammatory processes occurring after peripheral inflammation. On the other hand, besides their endocrine action, pro-inflammatory cytokines are mostly autocrine and paracrine factors that affect, in particular, cells synthesizing these mediators and nearby cells [33,34]. Subsequently, it could be hypothesized that the suppression of these locally synthesized pro-inflammatory cytokines in hypothalamic regions involved in GnRH-ergic activity may be also enough to release GnRH/LH secretion from the suppressory action of immune stress.

Therefore, in the present study, we aimed to determine the effect of the direct administration of an AChE inhibitor—neostigmine—into the third ventricle of the brain during GnRH/LH secretion in ewes during the follicular phase of the estrous cycle.

## 2. Results

### 2.1. Effect of Central Injection of Neostigmine and LPS Administration on LH, FSH, and Cortisol Releases

**Figure 1:**
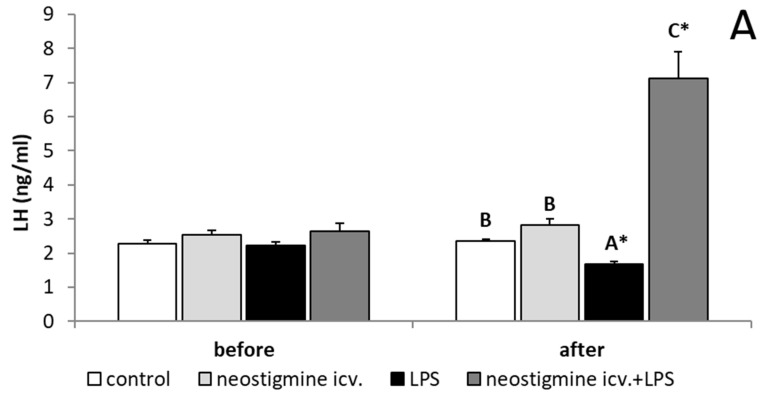

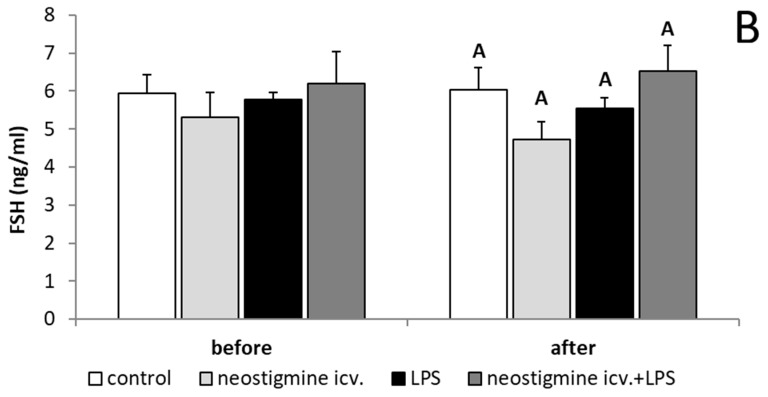
Effect of lipopolysaccharide (LPS; 400 ng/kg; intravenous) and neostigmine (1 mg/animal; intracerebroventricular (icv.)) injections on plasma concentration of luteinizing hormone (LH) (**A**) and follicle-stimulating hormone (FSH) (**B**) concentrations in the blood plasma. The data are presented as the mean value ± S.E.M. (*n* = 6 animals per group). All experiments were divided into two periods: a baseline with no treatment (2 to 0.5 h before) and the one after treatment (1 to 3 h after). *—asterisk indicates statistically significant differences between the baseline period and the period after treatment found during a Student’s *t*-test for dependent samples (“repeated measures”). A two-way ANOVA was used to analyze the concentrations of plasma hormones obtained only after treatment. Significant differences marked with different capital letters were analyzed by a two-way ANOVA followed by a Fisher’s post hoc test. Statistical significance was stated when *p* < 0.05.

**Figure 2:**
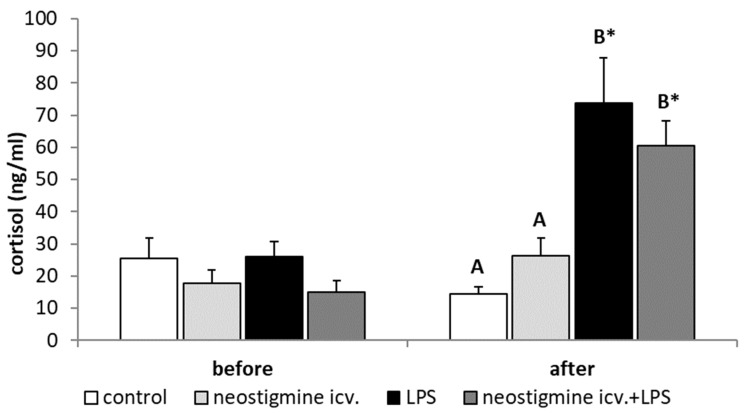
Effect of lipopolysaccharide (LPS; 400 ng/kg; intravenous) and neostigmine (1 mg/animal; intracerebroventricular (icv.)) injections on the concentration of cortisol in the blood plasma. The data are presented as the mean value ± S.E.M. (*n* = 6 animals per group). All experiments were divided into two periods: a baseline with no treatment (2 to 0.5 h before) and the one after treatment (1 to 3 h after). *—asterisk indicates statistically significant differences between the baseline period and the period after treatment found during a Student’s *t*-test for dependent samples (“repeated measures”). A two-way ANOVA was used to analyze the concentrations of plasma hormones obtained only after treatment. Significant differences marked with different capital letters were analyzed by a two-way ANOVA followed by a Fisher’s post hoc test. Statistical significance was stated when *p* < 0.05.

Endotoxin-induced inflammation reduced (*p* < 0.05) the plasma concentration of LH. Animals treated with LPS and centrally injected with neostigmine were characterized by an increased (*p* < 0.05) concentration of LH in comparison to all other groups (Figure 1A). In contrast, all treatments did not affect the plasma concentrations of FSH (Figure 1B). In all animals injected with LPS, increased (*p* < 0.05) plasma concentrations of cortisol were observed and these values were not influenced by the neostigmine treatment (Figure 2).

### 2.2. Effect of Central Injection of Neostigmine and LPS Administration on GnRHR Expression in the AP

**Figure 3:**
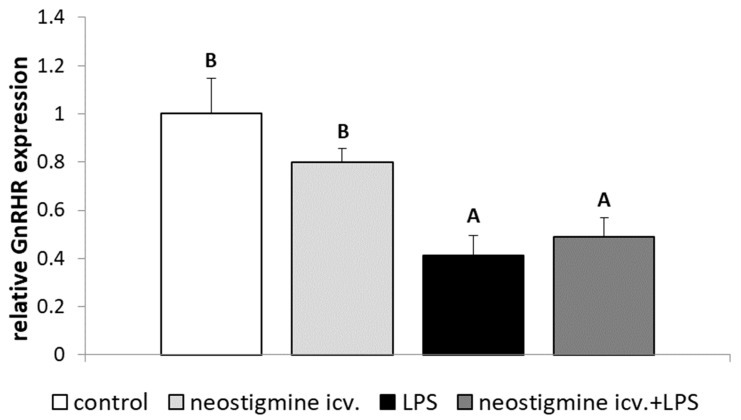
Effect of lipopolysaccharide (LPS; 400 ng/kg; intravenous) and neostigmine (1 mg/animal; intracerebroventricular (icv.)) injections on the relative protein expression (mean ± S.E.M.; *n* = 6 animals per group) of gonadotropin-releasing hormone receptor (GnRHR) in the anterior pituitary of ewes during the follicular phase of the estrous cycle. icv.—intracerebroventricular administration. The data are presented as the mean value ± S.E.M. (*n* = 6 animals per group). The results were analyzed using a two-way ANOVA. Significant differences marked with different capital letters were analyzed by a two-way ANOVA followed by a Fisher’s post hoc test. Statistical significance was stated when *p* < 0.05. The western blot bands representing the expression of GnRHR and ACTB protein are presented in Figure S1.

The inflammation caused by LPS injection decreased (*p* < 0.05) the expression of GnRHR in the AP. Central administration of neostigmine did not influence GnRHR protein expression in saline-treated ewes and failed to prevent an endotoxin-dependent decrease in this receptor expression in the AP (Figure 3).

### 2.3. Effect of Central Injection of Neostigmine and LPS Administration on GnRH, IL-1β, IL-6, TNFα, and IL-10 Contents in the POA

**Figure 4:**
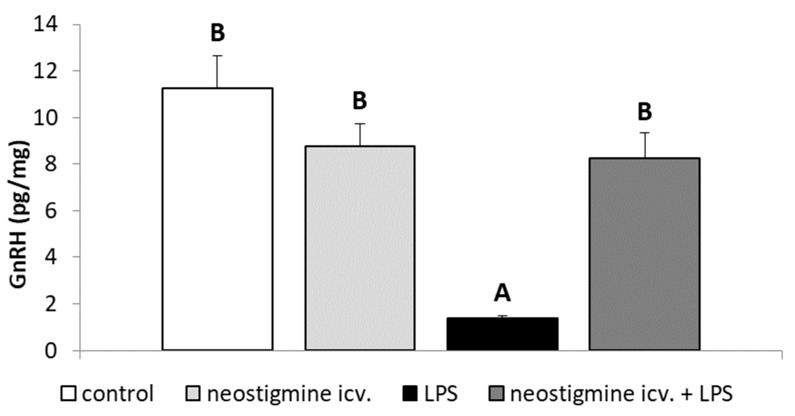
Effect of lipopolysaccharide (LPS; 400 ng/kg; intravenous) and neostigmine (1 mg/animal; intracerebroventricular (icv.)) injections on the content of gonadotropin-releasing hormone (GnRH) in the preoptic area of the hypothalamus in the ewes during the follicular phase of the estrous cycle. The data are presented as the mean value ± S.E.M. The results were analyzed using a two-way ANOVA. Significant differences marked with different capital letters were analyzed by a two-way ANOVA followed by a Fisher’s post hoc test. Statistical significance was stated when *p* < 0.05.

It was found that endotoxin treatment lowered (*p* < 0.05) the content of GnRH in the POA. Central injection of neostigmine remediated the inhibitory effect of LPS administration on the GnRH content in the POA (Figure 4).

**Figure 5:**
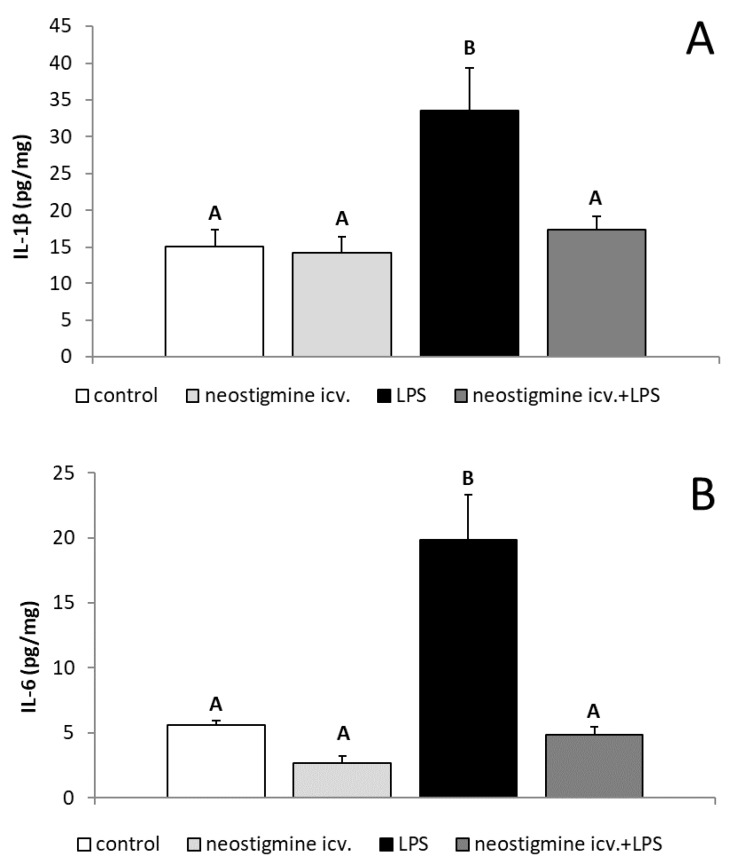

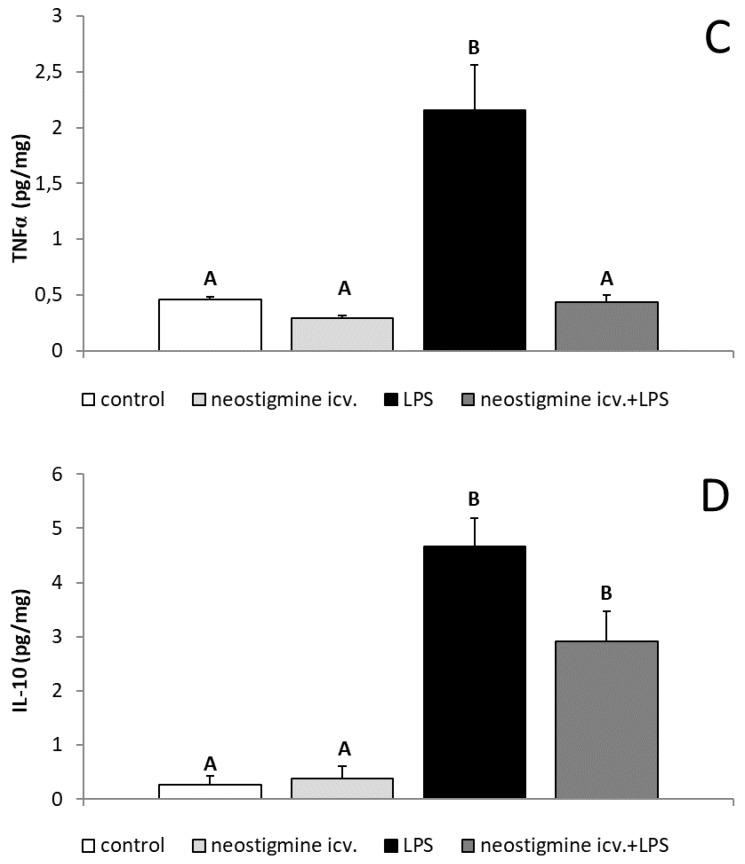
Effect of lipopolysaccharide (LPS; 400 ng/kg; intravenous) and neostigmine (1 mg/animal; intracerebroventricular (icv.)) injections on the level (mean ± S.E.M.; *n* = 6 animals per group) of pro-inflammatory cytokines: interleukin (IL)-1β (**A**), IL-6 (**B**), tumor necrosis factor (TNF)α (**C**), and IL-10 (**D**) in the preoptic area of the hypothalamus of ewes during the follicular phase of the estrous cycle. The data are presented as the mean value ± S.E.M. (*n* = 6 animals per group). The results were analyzed using a two-way ANOVA. Significant differences marked with different capital letters were analyzed by a two-way ANOVA followed by a Fisher’s post hoc test. Statistical significance was stated when *p* < 0.05.

The peripheral administration of bacterial endotoxin increased (*p* < 0.05) the content of cytokines, such as IL-1β, IL-6, TNFα, and IL-10 in the POA. The central injection of neostigmine prevented the LPS-induced increase (*p* < 0.05) in the level of all examined pro-inflammatory cytokines in this hypothalamic structure. On the other hand, the neostigmine treatment did not influence the production of anti-inflammatory IL-10 in the POA (Figure 5).

### 2.4. Effect of Central Injection of Neostigmine and LPS Administration on the Gene Expression of GnRH and Neuronal Acetylcholine Receptor Subunit Alpha-7 (CHRNA7) in the Hypothalamus

The injection of LPS decreased (*p* < 0.05) the gene expression of GnRH only in the ME but the central injection of neostigmine prevented this suppressive effect of endotoxin treatment. Moreover, in animals treated with neostigmine and LPS, an increased (*p* < 0.05) content of GnRH mRNA was found in the ME (Table 1).

It was also found that the central injection of neostigmine stimulated (*p* < 0.05) gene expression of CHRNA7 in all analyzed hypothalamic structures. Whereas, no effect of LPS treatment on the level of CHRNA7 mRNA in the hypothalamus was found (Table 2).

### 2.5. Effect of Central injection of Neostigmine and LPS Administration on the Gene Expression of GnRHR, LHβ, FSHβ, and CHRNA7 in the AP

Gene expression of GnRHR was suppressed (*p* < 0.05) by the administration of LPS but the central injection of neostigmine did not abolish the endotoxin-induced decrease in the GnRHR mRNA concentration in the AP. What is more, endotoxin treatment reduced (*p* < 0.05) LHβ mRNA expression in the AP but the central injection of neostigmine prevented this phenomenon. Neither neostigmine nor LPS influenced the FSHβ gene expression in the AP. It was found that the group of animals treated with neostigmine was characterized by a higher (*p* < 0.05) expression of mRNA CHRNA7 in comparison to control and LPS-treated groups. However, it was also determined that LPS injection significantly increased the gene expression of CHRNA7 in the AP but it was still lower than in neostigmine treated groups (Table 3).

## 3. Discussion

We have shown that central injection of neostigmine prevented an endotoxin-induced decrease in GnRH secretion in ewes during the follicular phase of the estrous cycle, which is in line with previous observations concerning peripherally administrated AChE-inhibitors [29,32]. We have noted that LPS treatment did not influence the gene expression of GnRH in the hypothalamic structures containing GnRH-ergic neurons perycaria, including POA, whereas it significantly decreased the concentration of GnRH peptide in this structure. Such finding supports the results of our previous study in which acute inflammation during the follicular phase of the estrous cycle suppressed GnRH secretion in the hypothalamus acting mainly on the posttranscriptional stages of GnRH synthesis [29,32]. The high stability of GnRH transcription in the follicular phase ewes generally corresponds with the characteristic of GnRH mRNA synthesis. It was previously reported that in GnRH neurons the amount of GnRH nuclear mRNA is only slightly lower than the amount of GnRH cytoplasmic mRNA [35,36]. Therefore, a greater amount of nuclear transcript allows for a steady flow of GnRH mRNA to the cytoplasm. This mRNA turnover, as well as rapid accumulation and fast degradation, are mainly responsible for changes in the content of GnRH mRNA in the perycarions. However, it should be stated that in our previous study on anestrous ewes it was shown that endotoxin-dependent inflammation decreased the expression of GnRH mRNA in the POA [5,37]. This suggests that circulating estradiol (during anestrous season the level of estradiol is presumably low) may have an effect on the inflammation of GnRH mRNA expression in the hypothalamus.

Moreover, we have found that endotoxin treatment decreased the amount of GnRH mRNA in the ME where GnRH-ergic neurons terminals are located. The central injection of neostigmine not only prevented this effect of inflammation but also together with LPS administration significantly increased the level of GnRH mRNA in the ME in comparison to the control ewes. This finding confirms the results of our previous studies suggesting that acute inflammation may reduce the axonal transport of GnRH mRNA from perycaria to the nerve terminals, thus reducing the amount of GnRH mRNA stored in the ME [29,32]. It is believed that the secretion of this decapeptide may be partially supported by the storage of the GnRH mRNA in nerve terminals. Therefore, the fact reported in our previous studies [29,32] that AChE inhibitors are capable of restoring the amounts of GnRH mRNA in the ME could have a profound positive influence on GnRH secretion. This may illustrate one of mechanisms via which AChE inhibitors do not allow for the suppression of GnRH/LH secretion during endotoxin-induced inflammation.

Although, there was no influence of inflammation on the expression of GnRH mRNA in the POA, it was found that the administration of endotoxin significantly reduced the content of GnRH peptide in this hypothalamic structure and the central injection of neostigmine prevented this effect of inflammation. The suppressory action of inflammation on GnRH release has been also reported in the study on ovariectomized ewes in which inflammation decreased the GnRH pulse amplitude without affecting the GnRH pulse frequency [38]. In our previous study, we also observed that LPS treatment induced decrease in the synthesis of GnRH peptide in the hypothalamus and that peripheral administration of AChE inhibitor—rivastigmine—not only reduced the suppressory effect of inflammation on the GnRH release but also could even stimulate this neurohormone secretion into the CSF of ewes in the follicular phase of the estrous cycle [32]. Moreover, in our later study on the peripheral administration of other AChE inhibitors—donepezil and neostigmine—the ability of these compounds to prevent an inflammatory-dependent decrease in GnRH synthesis in the POA in the follicular phase ewes was confirmed [29].

Our observations revealed that the effect of central neostigmine treatment on GnRH secretion was not a result of attenuation of the stress response. Treatment with neostigmine and LPS lead to a similar circulating concentration of cortisol to that determined in endotoxin-treated individuals. Cortisol, treated as a potent inhibitor of the reproductive process, could also reduce LH release in sheep [39]. It is worth mentioning that the components of the hypothalamic-pituitary-adrenal (HPA) axis, such as corticotropin-releasing hormone (CRH) and arginine vasopressin have been reported to inhibit the pulsatile GnRH and LH secretions [40]. However, the results of our study can be supported by the fact that the activation of the HPA axis has almost marginal influence on reproductive disorders during endotoxin-induced inflammatory challenges [39]. We have shown that the central injection of neostigmine prevented LPS-dependent increase in the synthesis of pro-inflammatory cytokines, such as IL-1β, IL-6, and TNFα in the POA, but on the other hand, it did not influence IL-10 synthesis in this hypothalamic structure. This anti-inflammatory effect of AChE inhibitor is based on the action of acetylcholine (ACh). It was found that the pharmacological inhibition of AChE activity increased the level of ACh. In turn, ACh acting through activation of CHRNA7 reduced the LPS-stimulated secretion of pro-inflammatory cytokines, including IL-1β, IL-6, and TNFα and decreased the expression of LPS-responsive receptors, such as CD14 and Toll-like receptor 4 [41,42,43]. In our study ACh also did not affect the synthesis of anti-inflammatory cytokine (IL-10). This fact confirms the inhibitory effect of ACh on pro-inflammatory cytokine production [43]. In neostigmine-treated ewes, the concentration of IL-10 in the POA was similar to that observed in animals treated solely with LPS. The anti-inflammatory action of ACh requires the expression of CHRNA7 in a target cell, an important element of the cholinergic anti-inflammatory pathway, since only the activation of this receptor counteracts cytokine release [43,44]. CHRNA7 is broadly expressed in the brain, also in the hypothalamus, and newer and newer data suggests that this receptor is necessary to protect the brain against neurodegenerative and neuroinflammatory processes [45]. The presence of CHRNA7 mRNA in hypothalamic structures as well as in the AP was also observed in our study. Moreover, the central injection of neostigmine stimulated the gene expression of CHRNA7 in the hypothalamus, which could result from increased stimulation of ACh. Our results broaden the knowledge suggesting the key role of pro-inflammatory cytokines in the central mechanism intruding the reproductive process during inflammatory state, particularly influencing GnRH secretion. It is suggested that IL-1β and TNFα acting centrally represent the major pro-inflammatory cytokines mediating the inflammation-dependent suppression of GnRH/LH secretion, whereas the role of IL-6 seems to be not important. In rats, the central administration of IL-1β to gonadetomized animals evoked a significant and prolonged decrease in plasma LH levels, whereas in intact cycling female rats, this decrease present on the afternoon of proestrus, resulted in the suppression of ovulation [46]. The inhibitory action of central IL-1β on the hypothalamic-pituitary unit activity was also found in the our previous studies [37,47] where in ewes the central IL-1β suppressed the synthesis of GnRH in the hypothalamus. However, it was suggested that in rats the ability of IL-1β to block ovulation could be, at least partially, related to opiate pathways activation [46]. Although the results of the study identifying the central pathway through which IL-1β influences the GnRH secretion in ewes suggested that this cytokine might down-regulate GnRH release via activation of NPY system, at the same time these results seemed to deny the involvement of catecholamines in the IL-1β-dependent inhibition of the GnRH secretion [47]. In rats, TNFα may be the other cytokine involved in the inhibition of HPG axis activity. It was found that the central administration of TNFα showed a suppressory effect on the GnRH/LH secretion equipotent to IL-1β, whereas IL-6 did not influence this process [14]. It is worth mentioning that accumulation of ACh in the hypothalamic tissue may also influence the GnRH secretion. In the ex vivo study on the hypothalamic and pituitary explants it was observed that ACh play a role in the control of LH secretion via stimulation of GnRH release from the hypothalamus [48]. The stimulatory action of ACh had been also confirmed in ex vivo study presenting capability of ACh to induce GnRH release from the rat MBH [49]. However, in a more recent study on rat hypothalamic neurons and GT1-7 line cells, the more intricate dependence between ACh and GnRH secretion was presented. This may result from the fact that ACh may modulate GnRH release and act through different cholinergic receptor subtypes to exert stimulatory and inhibitory effects [50]. On the one hand, the lack of changes in GnRH production in the hypothalamus after single central injection of neostigmine suggests that this treatment did not have a profound influence on this neurohormone secretion during the follicular phase of the estrous cycle. On the other hand, the sensitivity of the hypothalamic tissue and GnRH system on ACh action may be dependent upon physiological condition. Therefore, it cannot be unanimously stated that ACh does not influence the secretion of GnRH and/or LH in ewe during inflammation. In the previous in vitro study, LPS-treatment modulated the profile of ACh receptors, which modified the cell sensibility to the ACh action [51].

The neostigmine-induced changes in GnRH secretion in the hypothalamus influenced the LH secretion in ewes during the follicular phase of the estrous cycle. It was found that central injection of neostigmine prevented LPS-dependent decrease in the gene expression of LHβ in the AP. Unexpectedly, it was found that animals treated with neostigmine and LPS were characterized by an increased circulating concentration of LH even in comparison to the control ones. The ability of AChE inhibitors to reduce the influence of inflammation on LH secretion has been previously reported in studies on ewes [29,32]. Moreover, the peripheral administration of neostigmine also stimulated LH release after concomitant treatment with LPS [29]. This stimulatory effect of centrally administrated neostigmine on the LH secretion during inflammation seems to not simply result from changes in the GnRH secretion (neostigmine restored the synthesis of this neurohormone to the value noted in the control animals but not above it). Moreover, our results suggests that central neostigmine treatment failed to prevent an inflammatory-dependent decrease in the expression of GnRHR in the AP. Our proteomic analysis showed that the amount of GnRHR in this gland was similar in LPS-treated animals and in those receiving neostigmine. Because GnRHR expression influenced the sensitivity of the gland to the stimulation of GnRH [52], it could be supposed that in animals treated with neostigmine, the sensitivity of pituitary gonadotropin to the action of GnRH has been still reduced. In the course of inflammation, the reduced expression of GnRHR in the AP may be the effect of decreased secretion of the hypothalamic GnRH. This neurohormone, as one of the most potent regulators of its own receptor expression, launches the transcription of its receptor gene by many ways, including cAMP-, PKC-, and Ca^2+^-dependent signal transduction pathways [53]. It regulates the expression of its own receptor in the pituitary in a frequency-dependent manner. When GnRH is released in a pulsatile manner, it sustains the transcription of GnRHR mRNA as well as expression of GnRHR protein in the pituitary gonadotropes. While the continuous infusion of GnRH results in a desensitization of gonadotropes due to the reduction of the expression of GnRHR [52]. It can be suggested that an inflammatory-dependent decrease in the expression of GnRHR in the AP is not solely the effect of lowering GnRH secretion in the hypothalamus. Contrary, the current study proves that reduction of GnRHR expression in this gland is caused primarily by other factors present during inflammation. Pro-inflammatory cytokines and stress could be assigned to these factors because both IL-1β and CRH have been found to exhibit the suppressory action on the GnRHR expression [10,54,55]. Moreover, the expression of the pituitary GnRHR could be reduced directly by circulating endotoxin [6]. It could be supposed that during inflammation the central injection of neostigmine provoked the release of some factor or factors to the hypophyseal portal system, which stimulated LH release from the AP. The increase in ACh concentration in portal blood, which could reach the pituitary gland and influence gonadotrophs, seems to be the most obvious. However, in the in vitro study on AP cells from normal postpubertal female Sprague Dawley rats it was noted that although gonadotroph cells express multiple subtypes of functional ACh receptors, the secretory activities of both nicotinic ACh membrane receptor channels and muscarinic ACh membrane receptors are limited due to the co-activation of muscarinic acetylcholine receptor M4 and the generally inhibitory character of ACh action on LH secretion from gonadotroph cells [56]. Gamma-aminobutyric acid (GABA), which is co-released with ACh from many cells, including cholinergic forebrain neurons is also important [57]. It was previously found that GABA could be released from the ME to the hypophyseal portal blood system and could reach the pituitary [58]. Moreover, in the study on cultured female rat pituitary cells it was shown that GABA stimulates the release of LH [59]. However, further detailed studies are needed to identify these factors. Based on our short-term study, it is not possible to fully determine whether the observed increase in the LH secretion after neostigmine administration in endotoxin-treated animals is persistent or rather temporary. This issue requires future detailed long-term research as persistent increase in the circulating level of LH could have profound negative impact on fertility. It was previously reported that the high levels of gonadotropins (including LH) accompany premature ovarian failure [60]. Moreover, the increased LH/FSH ratio is observed in the course of PCOS. What is more, in this pathophysiological condition the increased androgen production by adrenal and theca cells is induced by common action of LH and hyperinsulinemia [21,61].

In summary, we have shown that the central administration of neostigmine might prevent the inflammatory-dependent decrease of GnRH/LH secretion in ewes and it had a stimulatory effect on LH release. This central action of neostigmine is largely due to its inhibition of the local pro-inflammatory cytokine synthesis in the hypothalamus, which indicates the importance of this mediators in the inhibition of GnRH secretion during the immune/inflammatory challenge. Moreover, we have noted that neostigmine action is targeted on the inhibition of pro-inflammatory cytokines because no effect of the treatment on the synthesis of anti-inflammatory IL-10 in the hypothalamus was found. In animals receiving, both, neostigmine and LPS, we have observed the increased plasma concentration of LH in comparison to the control group, which did not correspond with the changes in GnRH synthesis in the hypothalamus, may suggest that the central administration of the AChE inhibitor activated some additional central pathway leading to the stimulation of LH secretion. Also, it is intriguing whether the increase in LH secretion in endotoxin-treated individuals after central treatment with AChE inhibitor has a persistent or temporary character. Nevertheless, further studies are required to clarify these issues.

## 4. Materials and Methods

Detailed methodology of the study is included in the Supplementary Materials and Methods.

### 4.1. Animals and Experimental Procedures

In the study, 24 adult ewes during the reproductive season (September–October) were used. Their body condition was described as 3 in a five-point scale [62]. The animals were according to the recommendations of the National Research Institute of Animal Production [63]. One month before the experiment, ewes were cannulated with stainless steel guide cannulae (1.2 mm o.d.) into the third ventricle of the brain according to the method described elsewhere [64]. In order to standardize experimental conditions the stage of the estrous cycle of ewes was synchronized by a Chronogest^®^ CR (Merck Animal Health, Boxmeer, Netherlands) according to the method described in our previous study [29].

Animals (*n* = 24) were divided into four experimental groups (Table 4). The immune stress was induced in treated animals by the intravenous (iv.) injection of LPS from *Escherichia coli* 055:B5 (Sigma-Aldrich, St Louis, Missouri, USA) in a dose of 400 ng/kg, dissolved in saline (0.9% *w*/*v* NaCl) (Baxter, Deerfield, Illinois, USA) at a concentration of 10 mg/L. The dose of LPS was established and used in our previous studies [10,23,24,25,29,65]. Control animals received iv. injection of equivalent volume of saline. An intracerebroventricular (icv.) injection of neostigmine (1 mg/animal; Sigma-Aldrich, St. Louis, Missouri, USA) dissolved in 100 µL of Ringer’s solution through stainless steel catheter was performed 0.5 h before LPS/saline treatment. Control animals received only 100 µL of Ringer’s solution. The dose of neostigmine was chosen based on preliminary experiment, as the lowest dose which reduced the body temperature in endotoxin-treated animals. The ewes were euthanized 3 h after LPS/saline administration and the brains were rapidly removed from the skulls. The AP and four hypothalamic structures containing GnRH neurons such as preoptic area (POA), anterior hypothalamus (AHA), medial basal hypothalamus (MBH) and median eminence (ME) were dissected according to stereotaxic atlas of the sheep brain [66] as it was described elsewhere [32].

The experiment was conducted with the agreement of the Local Ethics Committee of Warsaw University of Life Sciences—SGGW (Warsaw, Poland; authorization no. 50/2013; date of approval: 18 September 2013).

### 4.2. Assays

#### 4.2.1. Radioimmunoassay of Hormones

The plasma LH concentration was assayed with a double-antibody radioimmunoassay (RIA) according to Stupnicki and Madej method [67]. The concentration of FSH was determined by double antibody RIA according to L’Hermite et al. [68]. The cortisol concentrations were determined according to Kokot and Stupnicki method [69].

#### 4.2.2. ELISA Assay for GnRH and Inflammatory Cytokines

The concentrations of GnRH in the POA homogenate was determined with a commercial GnRH ELISA kit (BlueGene Biotech CO., LTD., Shanghai, China) dedicated for sheep. The concentrations of IL-1β, IL-6, and TNFα in the POA were determined using a commercial IL-1β, IL-6, TNFα, and IL-10 ELISA kits (Cusabio Biotech Co. Ltd., Wuhan, China). The values of GnRH and inflammatory cytokines concentrations were normalized to total protein content in each sample assayed using Bradford method.

#### 4.2.3. Determining the Relative Gene Expression

A total RNA from the tissues were isolated using the components of a NucleoSpin^®^ RNA/Protein Kit (MACHEREY-NAGEL Gmbh & Co; Düren, Germany). A real-time RT-PCR was performed using HOT FIREPol EvaGreen^®^ qPCR Mix Plus (Solis BioDyne, Tartu, Estonia) in Rotor-Gene 6000 thermocycler (Qiagen, Duesseldorf, Germany). Specific primers for determining the expression of housekeeping genes and genes of interest were chosen on the basis of the results of our previous studies (Table 5). After the cycles, a final melting curve analysis was performed to confirm the specificity of the amplification.

Relative gene expression was calculated using the comparative quantification option [70] on a Rotor Gene 6000 software version 1.7 (Qiagen, Dusseldorf, Germany). Three housekeeping genes were examined: glyceraldehyde-3-phosphate dehydrogenase (GAPDH), β-actin (ACTB), and histone deacetylase 1 (HDAC1). The mean expression of these three housekeeping genes was used to normalize the expression of the analyzed genes. The results are presented in arbitrary units as the ratio of the target gene expression to the mean expression of the housekeeping genes.

#### 4.2.4. Western Blot Assay for GnRHR Expression in the AP

Before electrophoresis, the protein concentrations of samples isolated previously from the AP using the NucleoSpin^®^ RNA/Protein Kit (MACHEREY-NAGEL Gmbh & Co., Düren, Germany) were quantified using a Protein Quantification Assay Kit (MACHEREY-NAGEL Gmbh & Co., Düren, Germany). Downstream steps of the western blot assay of GnRHR protein expression in the AP was performed according to previously described method [29].

### 4.3. Statistical Analysis of Data

The results of hormones concentration are presented as the mean ± S.E.M. All experiments were divided into two parts: a baseline period with no treatment (2 to 0.5 h before) and a period after treatment (1 to 3 h after). To identify treatment effects, the mean values for the baseline and treatment periods were obtained. In order to compare the baseline period and a period after treatment, the Student’s T-test for dependent samples was used. Statistical significance was stated when *p* < 0.05.

The results of blood hormones concentration obtained only after treatment period, GnRH content in the POA and ME, pro-inflammatory cytokines concentration, GnRHR protein expression and all examined gene expressions were analyzed using a two-way ANOVA with two factors: inflammatory state and neostigmine treatment. Before ANOVA was conducted, the two assumptions were checked: normality (Shapiro–Wilk’s test) and homogeneity of the variances (Levene’s test). When a significant treatment by time interaction was observed, the Fisher’s least significant difference post hoc test was used to compare pre- with post-treatment values. Statistical significance was defined as *p* < 0.05.

The statistical analysis was performed using a STATISTICA 10 software (StatSoft Inc., Tulsa, OK, USA).

## Figures and Tables

**Table 1:** Effect of lipopolysaccharide (LPS; 400 ng/kg; intravenous) and neostigmine (1 mg/animal; intracerebroventricular (icv.)) administration on the relative mRNA expression (mean ± S.E.M.; *n* = 6 animals per group) of gonadotropin-releasing hormone (GnRH) in the hypothalamus of ewes in the follicular phase. POA—the preoptic area; AHA—the anterior hypothalamus; MBH—the medial basal hypothalamus; ME—the median eminence; control—group treated with saline. In the case of all examined hypothalamic structures GnRH mRNA expression data were normalized to the average relative level of this mRNA expression in the control ewes, which was set to 1.0. Significant differences marked with different capital letters were analyzed by a two-way ANOVA followed by a Fisher’s post hoc test.

Structure	GnRH Relative Gene Expression			
	Control	Neostigmine icv.	LPS	Neostigmine icv. + LPS
POA	1 ± 0.2 ^A^	0.9 ± 0.2 ^A^	0.6 ± 0.2 ^A^	0.7 ± 0.2 ^A^
AHA	1 ± 0.1 ^A^	0,9 ± 0.1 ^A^	1 ± 0.1 ^A^	1 ± 0.2 ^A^
MBH	1 ± 0.1 ^A^	0.9 ± 0.2 ^A^	1 ± 0.1 ^A^	1.2 ± 0.2 ^A^
ME	1 ± 0.1 ^B^	1 ± 0.1 ^B^	0.5 ± 0.2 ^A^	1.7 ± 0.2 ^C^

**Table 2:** Effect of lipopolysaccharide (LPS; 400 ng/kg; intravenous) and neostigmine (1 mg/animal; intracerebroventricular (icv.)) administrations on the relative mRNA expression (mean ± S.E.M.; n = 6 animals per group) of cholinergic receptor nicotinic alpha 7 subunit (CHRNA7) in the hypothalamus of ewes in the follicular phase. POA—the preoptic area; AHA—the anterior hypothalamus; MBH—the medial basal hypothalamus; ME—the median eminence; control—group treated with saline. In the case of all examined hypothalamic structures gene expression data were normalized to the average relative level of this mRNA expression in the control ewes, which was set to 1.0. Significant differences marked with different capital letters were analyzed by a two-way ANOVA followed by a Fisher’s post hoc test.

Structure	CHRNA7 Relative Gene Expression			
	Control	Neostigmine icv.	LPS	Neostigmine icv. + LPS
POA	1 ± 0.2 ^A^	1.7 ± 0.2 ^B^	1.3 ± 0.1 ^A^	1.7 ± 0.1 ^B^
AHA	1 ± 0.1 ^A^	1.3 ± 0.1 ^B^	0.9 ± 0.1 ^A^	1.3 ± 0.1 ^B^
MBH	1 ± 0.1 ^A^	1.4 ± 0.1 ^B^	0.9 ± 0.1 ^A^	1.3 ± 0.1 ^B^
ME	1 ± 0.1 ^A^	2.2 ± 0.3 ^B^	1 ± 0.1 ^A^	1.8 ± 0.2 ^B^

**Table 3:** Effect of lipopolysaccharide (LPS; 400 ng/kg; intravenous) and neostigmine (1 mg/animal; intracerebroventricular (icv.)) administrations on the relative mRNA expression (mean ± S.E.M.; n = 6 animals per group) of gonadotropin-releasing hormone receptor (GnRHR), luteinizing hormone β-subunit (LHβ), follicle-stimulating hormone β-subunit (FSHβ), and cholinergic receptor nicotinic alpha 7 subunit (CHRNA7) genes in the anterior pituitary of ewes in follicular phase. POA—the preoptic area; AHA—the anterior hypothalamus; MBH—the medial basal hypothalamus; ME—the median eminence; control—group treated with saline. In the case of all examined genes, the mRNA expression data were normalized to the average relative level of this mRNA expression in the control ewes, which was set to 1.0. Significant differences marked with different capital letters were analyzed by a two-way ANOVA followed by a Fisher’s post hoc test.

Gene	Anterior Pituitary			
	Control	Neostigmine icv.	LPS	Neostigmine icv. + LPS
GnRHR	1 ± 0.1 ^BC^	1.2 ± 0.1 ^C^	0.5 ± 0.1 ^A^	0.7 ± 0.1 ^AB^
LHβ	1 ± 0.1 ^B^	1.3 ± 0.2 ^B^	0.5 ± 0.1 ^A^	1 ± 0.1 ^B^
FSHβ	1 ± 0.3 ^A^	1 ± 0.3 ^A^	1.1 ± 0.2 ^A^	0.8 ± 0.2 ^A^
CHRNA7	1 ± 0.3 ^A^	6.8 ± 1.8 ^C^	2.4 ± 0.6 ^B^	4.8 ± 0.8 ^C^

**Table 4:** Scheme of the experiment.

Group No.	Group Name	No. of Animals	Experimental Treatment I (icv.)	Dose (mg/Animal)	Experimental Treatment II (iv.)	Dose (ng/kg)
1	Control	6	Ringer’s solution	0	NaCl	0
2	Neostigmine-treated	6	Neostigmine	1	NaCl	0
3	LPS-treated	6	Ringer’s solution	0	LPS	400
4	Neostigmine- + LPS-treated	6	Neostigmine	1	LPS	400
Total number of animals		24				

**Table 5:** List of full names and abbreviations of all genes analyzed by Real-Time PCR.

GenBank Acc. No.	Gene	Amplicon Size (bp)	Forward/Reverse	Sequence 5′→3′	Reference
NM_001034034	*GAPDH* *glyceraldehyde-3-phosphate dehydrogenase*	134	forward	AGAAGGCTGGGGCTCACT	[24]
			reverse	GGCATTGCTGACAATCTTGA	
U39357	*ACTB* *beta actin*	168	forward	CTTCCTTCCTGGGCATGG	[24]
			reverse	GGGCAGTGATCTCTTTCTGC	
BC108088.1	*HDAC1* *histone deacetylase1*	115	forward	CTGGGGACCTACGGGATATT	[24]
			reverse	GACATGACCGGCTTGAAAAT	
NM_001009397	*GnRHR* *gonadotropin-releasing hormone receptor*	150	forward	TCTTTGCTGGACCACAGTTAT	[32]
			reverse	GGCAGCTGAAGGTGAAAAAG	
U02517	*GnRH* *gonadotropin-releasing hormone*	123	forward	GCCCTGGAGGAAAGAGAAAT	[32]
			reverse	GAGGAGAATGGGACTGGTGA	
X52488	*LHB* *luteinizing hormone beta-subunit*	184	forward	AGATGCTCCAGGGACTGCT	[32]
			reverse	TGCTTCATGCTGAGGCAGTA	
X15493	*FSHB* *follicle stimulating hormone beta-subunit*	131	forward	TATTGCTACACCCGGGACTT	[32]
			reverse	TACAGGGAGTCTGCATGGTG	
BC_149340	*CHRNA7* *neuronal acetylcholine receptor subunit alpha-7*	114	forward	TGGAAGCCAGACATTCTCCT	[25]
			reverse	GATGCCTGGAGGGAGGTACT	

## Supplementary Materials

Supplementary materials can be found at https://www.mdpi.com/1422-0067/20/18/4598/s1.
