# Supplementary material for: Effect of Central Injection of Neostigmine on the Bacterial Endotoxin Induced Suppression of GnRH/LH Secretion in Ewes during the Follicular Phase of the Estrous Cycle
Source: Int J Mol Sci. 2019 Sep 17;20(18):4598. doi: 10.3390/ijms20184598 (PMC6769544; doi:10.3390/ijms20184598)
Supplement: Supplementary file 1 [file ijms-20-04598-s001.pdf]

Supplementary Materials and Methods

Animals and experimental procedures

In the study were used 24 adult, 2-year-old Blackhead ewes during the reproductive season (September–October). The conditions of animal maintenance were good (body condition was described as 3 in a five-point scale) [62]. Animals were adapted to experimental conditions for three months. In order to prevent isolation stress animals had a permanent visual contact. The animals were fed a constant diet of commercial concentrates with hay and water available *ad libitum*, according to the recommendations of the National Research Institute of Animal Production for adult ewes [63]. One month before the experiment, ewes were cannulated with stainless steel guide cannulae (1.2 mm o.d.) into the third ventricle of the brain according to the method described elsewhere [64]. In order to standardize experimental conditions the stage of the estrous cycle of ewes was synchronized by a Chronogest® CR (Merck Animal Health, Boxmeer, Netherlands) method using an intra-vaginal sponge impregnated with 20 mg of a synthetic progesterone-like hormone. All ewes had Chronogest® CR sponges placed for 14 days. Following sponge removal, the ewes received an intramuscular injection of 500 iu pregnant mare's serum gonadotropin (PMSG) (Merck Animal Health, Boxmeer, the Netherlands). The experimental procedure was performed 24 h following PMSG injection.

All ewes had venous catheters implanted into their jugular vein the one day before the start of the experiment. Animals ($n = 24$) were randomly divided into four experimental groups (Table 4). In treated animals, the immune stress was induced by the intravenous (iv.) injection of LPS from *Escherichia coli* 055:B5 (Sigma-Aldrich, St Louis, MO, USA) in a dose of 400 ng/kg, dissolved in saline (0.9% w/v NaCl) (Baxter, Deerfield, IL, USA) at a concentration of 10 mg/l. The dose of LPS was established and used in our previous studies [10,23,24,25,29,65]. Control animals received iv. injection of equivalent volume of saline. An intracerebroventricular (icv.) injection of neostigmine (1 mg/animal; Sigma-Aldrich, St. Louis, MO, USA) dissolved in 100 µl of Ringer's solution through stainless steel catheter was performed 0.5 h before LPS/saline treatment. Control animals received only 100 µl of Ringer's solution. The animals were euthanized 3 h after LPS/saline administration and the brains were rapidly removed from the skulls. The AP and four hypothalamic structures involved in the GnRH-ergic activity such as preoptic area (POA), anterior hypothalamus (AHA), the medial basal hypothalamus (MBH) and median eminence (ME) were dissected according to stereotaxic atlas of the sheep brain [66] as it was described elsewhere [32]. All tissues were frozen immediately after collection in liquid nitrogen and then stored at −80 °C.

The experiment was conducted with the agreement of the Local Ethics Committee of Warsaw University of Life Sciences – SGGW (Warsaw, Poland; authorization no. 50/2013; date of approval: 2013, September 18).

Assays

Radioimmunoassay for LH

The plasma LH concentration was assayed with a double-antibody radioimmunoassay (RIA) using anti-ovine-LH and anti-rabbit-γ-globulin antisera and ovine standard (teri.oLH, Tucker Endocrine Research Institute), according to Stupnicki and Madej method [67]. The assay sensitivity was 0.3 ng/ml and the intra- and inter-assay coefficients of variation were 9% and 12.5%, respectively.

Radioimmunoassay for follicle-stimulating hormone (FSH)

The concentration of FSH was determined by double antibody RIA using anti ovine-FSH (teri.anti-oFSH) and anti-rabbit-γ-globulin antisera, according to L'Hermite et al. [68]. The anti-FSH, as well as the FSH standard (teri. oFSH-and teri. FSH ig), were kindly supplied by Dr. L.E. Reichert Jr. (Tucker Endocrine Research Institute LLC, Atlanta, Georgia, USA). The assay sensitivity was 1.5 ng/ml and the intra- and inter-assay coefficients of variation were 3.5% and 11.3%, respectively.

Radioimmunoassay for cortisol

The cortisol concentrations were determined by RIA according to Kokot and Stupnicki method [69], using rabbit anti-cortisol antisera (R/75) and an HPLC-grade cortisol standard (Sigma-Aldrich, St. Louis, MO, USA). The assay sensitivity was 1 ng/ml and the intra- and inter-assay coefficients of variation were 9% and 12%, respectively.

ELISA assay for the GnRH and inflammatory cytokines

The POA tissues were homogenized in 500 μl of phosphate buffered saline (0.02 M) (. Then homogenates were subjected to two freeze-thaw cycles to further break the cell membranes. After that, the homogenates were centrifuged for 15 min at 1500 x g in 4 °C. The supernatants were aliquoted and stored until assay in −80 °C. The concentrations of GnRH in the POA were determined with a commercial GnRH ELISA kit (BlueGene Biotech CO., LTD. China) dedicated for sheep. The concentrations of IL-1β, IL-6, and TNFα in the POA were determined using a commercial IL-1β, IL-6, TNFα and IL-10 ELISA kits (Cusabio Biotech Co. Ltd., Wuhan, China) designed and validated for sheep. All stages of ELISA analysis were performed according manufacturer's protocol. The incubation of plates and absorbance measurement at 450 nm were performed using a VersaMax reader (Molecular Devices LLC., Sunnyvale, California, United States). The sensitivity of GnRH assays was 1.0 pg/ml (cat no. E14G0213). The sensitivities of assays for inflammatory cytokines were 3.9 pg/ml (IL-1β; cat no. CSB-E10115Sh), 2 pg/ml (IL-6; cat no. CSB-E10116Sh), 3.12 pg/ml (TNFα; CSB-E13853Sh) and 5 pg/ml (IL-10; CSB-E12817Sh). The values of GnRH and inflammatory cytokines concentrations were normalized to total protein content in each sample assayed using Bradford method.

Determining the relative gene expression

A total RNA from the hypothalamic structures and the AP were isolated using the components of a NucleoSpin® RNA/Protein Kit (MACHEREY-NAGEL Gmbh & Co; Düren, Germany) according to a manufacturer's instruction. The purity and concentration of isolated RNA were spectrophotometrically quantified by measuring the optical density at 230, 260 and 280 nm in a NanoDrop 1000 instrument (Thermo Fisher Scientific Inc., Waltham, USA). The RNA integrity was verified by electrophoresis using 1% agarose gel stained with ethidium bromide (Sigma-Aldrich, St. Louis, MO, USA). A Maxima™ First Strand cDNA Synthesis Kit for RT-qPCR (Thermo Fisher Scientific Inc., Waltham, USA) was used to prepare cDNA synthesis. For this PCR synthesis 1 μg of total RNA was used as a starting material.

A real-time RT-PCR was performed using HOT FIREPol EvaGreen® qPCR Mix Plus (Solis BioDyne, Tartu, Estonia) components and HPLC-grade oligonucleotide primers synthesised by Genomed (Poland). Specific primers for determining the expression of housekeeping genes and genes of interest were chosen on the basis of the results of our previous studies (Table 5). One tube contained: 4 μl PCR Master Mix (5x), 14 μl RNase-free water, 1 μl primers (0.5 μl each, working concentration 0.25 μM) and 1 μl cDNA template. The tubes were run on a Rotor-Gene 6000 (Qiagen, Duesseldorf, Germany). The following protocol was used: 15 min in 95 °C for activating Hot Star DNA polymerase and finally the PCR including 30 cycles at 10 sec in 95 °C for denaturation, 20 sec in 60 °C for annealing, and 10 sec in 72 °C for extension. After the cycles, a final melting curve analysis under continuous fluorescence measurements was performed to confirm the specificity of the amplification.

Relative gene expression was calculated using the comparative quantification option [70] on a Rotor Gene 6000 software version 1.7 (Qiagen, Dusseldorf, Germany). Three housekeeping genes were examined: glyceraldehyde-3-phosphate dehydrogenase (GAPDH), β-actin (ACTB) and histone deacetylase 1 (HDAC1). The mean expression of these three housekeeping genes was used to normalise the expression of the analysed genes. The results are presented in arbitrary units, as the ratio of the target gene expression to the mean expression of the housekeeping genes.

Western blot assay for GnRHR expression in the AP

Western blot assay of GnRHR protein expression in the AP was performed according to previously described method [29]. Before electrophoresis, the protein concentrations of samples isolated previously from the AP using the NucleoSpin® RNA/Protein Kit (MACHEREY-NAGEL Gmbh & Co., Düren,

Germany) were quantified using a Protein Quantification Assay Kit (MACHEREY-NAGEL Gmbh & Co., Düren, Germany). The appropriate volume of a molecular grade water (Sigma-Aldrich, St. Louis, MO, USA) was added to a volume of sample containing 50 µg of total protein to bring the total sample volume to 20 µl. Next, 19 µl of a Laemmli buffer (Sigma-Aldrich, St. Louis, MO, USA) and 1 µl of β–mercaptoethanol (Sigma-Aldrich, St. Louis, MO, USA) were added. Such mixtures were boiled for 3 min. Electrophoresis was then performed in the presence of molecular weight markers (Spectra Multicolor Broad Range Protein Ladder, Thermo Fisher Scientific Inc., Waltham, MA, USA). Denatured samples and molecular weight standards were loaded onto 4–12% polyacrylamide gels and subjected to electrophoresis in a Tris-glycine running buffer using the Protean II xi Cell (Bio-Rad Laboratories, Inc., Hercules, CA, USA) according to the manufacturer's instructions. Next, proteins were transferred in a Tris-glycine blotting buffer to polyvinylidene difluoride membranes (Immobilon™-P (0.45 µm), Merck KGaA, Darmstadt, Germany) using a Trans-Blot® SD Semi-Dry Transfer Cell (Bio-Rad Laboratories, Inc., Hercules, CA, USA) for 30 min at 20 V. The membranes were blocked for 1 h at a room temperature in a blocking buffer made up of Tris buffered saline at pH 7.5 with 0.05% Tween-20 (TBST) (Sigma-Aldrich, St. Louis, MO, USA) containing 3% bovine serum albumin fraction V (Sigma-Aldrich, St. Louis, MO, USA). Next, membranes were incubated overnight at 4 °C with the following primary antibodies: goat anti-GnRHR polyclonal antibody (cat no. sc-8682, Santa Cruz Biotechnology Inc., Dallas, USA), and mouse anti-ACTB monoclonal antibody (cat no. sc-47778, Santa Cruz Biotechnology Inc., Dallas, USA) dissolved in blocking buffer at dilutions of 1:500 and 1:1000, respectively. After washing three times, membranes were incubated with the following secondary HRP conjugated antibodies: donkey anti-goat IgG-HRP (cat no. sc-2304, Santa Cruz Biotechnology Inc., Dallas, TX, USA) and goat anti-mouse IgG1 heavy chain (HRP) (cat no. ab97240, Abcam, Cambridge, UK) dissolved in blocking buffer at a dilution of 1:10,000. After washing three times, the membranes were visualised using chromogenic detection with a Pierce 1-step TMB-blotting substrate solution (Thermo Fisher Scientific, Waltham, MA, USA). After visualisation, the membranes were dried and scanned using an EPSON Perfection V370 Photo scanner (Seiko Epson Corporation, Suwa, Japan). Densitometric analysis of the scanned membrane was performed using a software ImageJ (Research Services Branch, National Institute of Mental Health, Bethesda, MD, USA).

GnRHR (36 kDa)

ACTB (42 kDa)

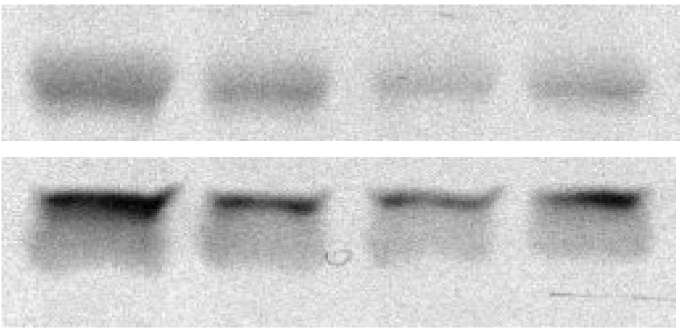

control *neostigmine icv.* *LPS* *neostigmine icv. + LPS*

Figure S1. The western blot bands represent the expression of GnRHR and ACTB protein expression in the pooled samples of anterior pituitaries of ewes (n = 6) during the follicular phase of the estrous cycle. *control* – ewes treated with saline; *neostigmine icv.* – animals treated with neostigmine (1 mg/animal; intracerebroventricular (icv.)); LPS – ewes injected with lipopolysaccharide (LPS; 400 ng/kg; intravenous); *neostigmine icv. + LPS* – animals injected with and neostigmine and LPS.

Statistical analysis of data

The results of hormones concentration are presented as the mean ± S.E.M. All experiments were divided into two parts: a baseline period with no treatment (2 to 0.5 h before) and a period after treatment (1 to 3 h after). To identify treatment effects, the mean values for the baseline and treatment periods were obtained. In order to compare the baseline period and a period after treatment, the

Student's T-test for dependent samples ('repeated measures') was used. Statistical significance was stated when $p < 0.05$.

The results of blood hormones concentration obtained only after treatment period, GnRH content in the POA and ME, pro-inflammatory cytokines concentration, GnRHR protein expression and all examined genes expressions were analysed using a two-way ANOVA with two factors: inflammatory state and neostigmine treatment. Before ANOVA was conducted, it's two assumptions were checked: normality (Shapiro-Wilk's test) and homogeneity of the variances (Levene's test). When a significant treatment by time interaction was observed, the Fisher's least significant difference *post hoc* test was used to compare pre- with post-treatment values. Statistical significance was defined as $p < 0.05$.

The statistical analysis was performed using a STATISTICA 10 software (StatSoft Inc., Tulsa, OK, USA).
